# Supplementary material for: Analogs of Periplanetasin-4 Exhibit Deteriorated Membrane-Targeted Action
Source: J Microbiol Biotechnol. 2020 Mar 11;30(3):382–90. doi: 10.4014/jmb.1912.12044 (PMC9728181; doi:10.4014/jmb.1912.12044)
Supplement: Supplementary file 1 [file JMB-30-3-382-supple.pdf]

J. Microbiol. Biotechnol. (2020), 30(3), 382–390
https://doi.org/10.4014/jmb.1912.12044

jmb

Analogs of Periplanetasin-4 Exhibit Deteriorated Membrane-Targeted Action S

Heejeong Lee[1†], Jae Sam Hwang[2†], and Dong Gun Lee[1*]

[1]School of Life Sciences, BK21 Plus KNU Creative BioResearch Group, Kyungpook National University, Daegu 41566, Republic of Korea
[2]Department of Agricultural Biology, National Academy of Agricultural Science, RDA, Wanju 55365, Republic of Korea

Received: December 27, 2019
Revised: March 5, 2020
Accepted: March 10, 2020

First published online:
March 11, 2020

**Corresponding author*
Phone: +82-53-950-5373
Fax: +82-53-955-5522
E-mail: dglee222@knu.ac.kr

[†]These authors contributed equally to this work.

Supplementary data for this paper are available on-line only at http://jmb.or.kr.

pISSN 1017-7825, eISSN 1738-8872

Copyright© 2020 by
The Korean Society for Microbiology and Biotechnology

Periplanetasin-4 is an antimicrobial peptide with 13 amino acids identified in cockroaches. It has been reported to induce fungal cell death by apoptosis and membrane-targeted action. Analogs were designed by substituting arginine residues to modify the electrostatic and hydrophobic interactions accordingly and explore the effect of periplanetasin-4 through the increase of net charge and the decrease of hydrophobicity. The analogs showed lower activity than periplanetasin-4 against gram-positive and gram-negative bacteria. Similar to periplanetasin-4, the analogs exhibited slight hemolytic activity against human erythrocytes. Membrane studies, including determination of changes in membrane potential and permeability, and fluidity assays, revealed that the analogs disrupt less membrane integrity compared to periplanetasin-4. Likewise, when the analogs were treated to the artificial membrane model, the passage of molecules bigger than FD4 was difficult. In conclusion, arginine substitution could not maintain the membrane disruption ability of periplanetasin-4. The results indicated that the attenuation of hydrophobic interactions with the plasma membrane caused a reduction in the accumulation of the analogs on the membrane before the formation of electrostatic interactions. Our findings will assist in the further development of antimicrobial peptides for clinical use.

Keywords: Periplanetasin-4, *Periplaneta americana*, arginine substitution, membrane disruption, liposome

Introduction

Humanity continues to encounter and overcome endless challenge in an ongoing struggle for survival. Food shortages due to growing populations might be ameliorated by sourcing from the largest and most widely distributed group of animals in the world, namely, insects. Insect diversity can be attributed to an incredible variety of mechanisms and biological process regulation [1]. Indeed, the utilization of such organisms, which contain a massive quantity of protein, has been considered in various fields as a way to solve food problems [2, 3]. Unfortunately, the potential introduction of insects as food could be jeopardized by a shared aversion towards consuming insects [4]. Thus, an alternative approach to the use of insects has been suggested. Insects that can survive in various environments have a well-established defense system against external pathogens. Researchers are trying to capitalize on this feature for the purpose of other survival issues related to public health. The sustainable proteins found in insects can be major sources of antimicrobial peptides (AMPs) [2, 3].

Naturally, novel AMPs can be purified and identified from the bacteria-induced hemolymph in insects [3, 5–7]. Immunological agents such as hormones and peptides, including neuropeptides and AMPs, can be obtained from the immune systems of insects [1]. In a previous study, we isolated periplanetasin-4 from the American cockroach, one of the insects considered most disgusting. Periplanetasin-4 is a cationic peptide with low hemolysis and potent antimicrobial activity that mainly induces membrane damage and apoptosis [6, 8]. The structure–function relationship of AMPs suggests that a number of parameters modulate

antimicrobial activity, including the charge distribution, net positive charge, amphipathicity, and helical propensity [9]. To improve antibacterial activity and/or toxicity towards host cells, known peptide sequences can be modified, often by single amino acid substitution. These modifications affect relevant biophysical properties such as the hydrophobic/polar residue balance, net charge and the resulting amphipathicity, and/or the tendency for self-aggregation. All of these features are closely linked to peptide activity and/or cell selectivity [9–11]. Herein, periplanetasin-4 was designed with altered electrostatic and hydrophobic properties. Three analogs were synthesized and their differences in antibacterial activity were investigated.

Materials and Methods

Peptide Synthesis

All peptides were chemically synthesized by Anygen (Korea), using the solid-phase peptide synthesis method with Fmoc (9-fluorenyl-methoxycarbonyl) chemistry. Periplanetasin-4 and its analogs were manually synthesized. Assembly of the peptides was achieved with a 60-min cycle for each residue at ambient temperature using a reactor with a specially-designed shape. The crude peptide was repeatedly washed with diethyl ether and dissolved in 0.1 mM ammonium bicarbonate, water and acetonitrile. The mixture was then freeze-dried in a lyophilizer after the salts were excluded. The peptides were purified using preparative reverse-phase high-performance liquid chromatography (RP-HPLC) on C_{18} columns (20 × 250 mm; Shim-pack; Shimadzu, Japan). The purity of the peptides was verified with analytical RP-HPLC, and the peptide masses were confirmed using matrix-assisted laser desorption ionization time-of-flight mass spectrometry (MALDI-TOF MS; Shimadzu). The course of the reaction was monitored using HPLC. The purity of the periplanetasin-4 and the three analogs was >90%.

Peptide Characterization

Analytical and preparative reverse-phase HPLC runs were performed with a Shimadzu 20A or 6A gradient system. Data were collected using an SPD-20A detector at 230 nm. Chromatographic separations were achieved with a 1%/min linear gradient of buffer B in A (A = 0.1% TFA in H_2O; B = 0.1% TFA in acetonitrile (CH_3CN)) over 40 min at flow rates of 1 and 8 ml/min using Shimadzu C_{18} analytical (5 µm, 0.46 cm × 25 cm) and preparative C_{18} (10 µm, 2.5 cm × 25 cm) columns, respectively. The three analogs were, Per[Arg^9] called Anal-1, Per[$Arg^{8,9}$] called Anal-2, and Per[$Arg^{3,8,9}$] called Anal-3. As shown in Fig. S1, the HPLC retention times (min) for periplanetasin-4, Anal-1, Anal-2, and Anal-3 were 19.975, 14.419, 12.644 and 12.646, respectively. Mass spectrometry was also performed. The observed masses of periplanetasin-4, Anal-1, Anal-2, and Anal-3 were 1,503.8, 1,556.88, 1,549.89, and 1,568.94, respectively.

Bacterial Strains and Antibacterial Susceptibility Test

Enterococcus faecium (ATCC 19434), *Enterococcus faecalis* (ATCC 29212), *Pseudomonas aeruginosa* (ATCC 27853), and *Salmonella enteritidis* (ATCC 13076) were obtained from the American Type Culture Collection (ATCC; USA). *Staphylococcus epidermidis* (KCTC 1917), *Streptococcus mutans* (KCTC 3065) and *Salmonella typhimurium* (KCTC 1926) were obtained from the Korean Collection for Type Cultures (KCTC). *Escherichia coli* (BW25113) was obtained from the Coli Genetic Stock Center. Bacterial strains were cultured in LB broth (BD) at 37°C with aeration. The antimicrobial activity of AMPs against microbial pathogens was determined using the Clinical and Laboratory Standards Institute method as previously described [7]. After 24 h of incubation, growth was measured using the microtiter BioTek ELx800 Absorbance Reader (BioTek Instruments, USA) by monitoring the absorption at 600 nm.

Hemolytic Effect Against Human Erythrocytes

Fresh human erythrocytes were centrifuged at 2,000 ×*g* for 10 min and washed three times with phosphate-buffered saline (PBS: 35 mM phosphate buffer/150 mM NaCl, pH 7.4). The final concentration of erythrocytes was 4%. The erythrocyte suspension was transferred to sterilized 96-well plates and two-fold serial dilutions of the peptides were added to the wells of a 96-well plate. The samples were then incubated with the compounds at 37°C for 1 h and the plate was centrifuged at 1,500 ×*g* for 10 min. An aliquot of the supernatant was taken, and then, the hemolytic activity of the compounds was evaluated by measuring the release of hemoglobin from a 4% suspension of human erythrocytes at 414 nm with an ELISA reader. Hemolytic levels of zero and 100% were determined in PBS alone and with 0.1 Triton X-100, respectively. The hemolysis percentage was calculated with the following equation: hemolysis (%) = [(Abs_{414nm} in the peptide solution - Abs_{414nm} in PBS)/(Abs_{414nm} in 0.1% Triton X-100 - Abs_{414nm} in PBS)] × 100 [7].

Measurement of Changes in Membrane Potential

The effects of the peptides on the membrane potential of *E. coli* were determined using the membrane potential-sensitive fluorescent dye 3,3′-dipropylthiadicarbocyanine iodide [$DiSC_3(5)$]. The cells were harvested and suspended to an OD_{600} of 0.05 in 5 mM of HEPES (pH 7.2) with 20 mM of glucose. The fluorescence of the probes was monitored using a Shimadzu at an excitation wavelength of 622 nm and emission wavelength of 670 nm. After reaching the maximum uptake of the dye by bacteria, which was indicated by a minimum in fluorescence, peptide solution was added to the cells and the membrane depolarization was monitored based on the increase in fluorescence [12–14].

To analyze membrane permeabilization after treatment with the peptides, the cells were suspended in PBS and incubated for 2 h at

37°C. After incubation, the cells were harvested by centrifugation and resuspended in PBS. Subsequently, the cells were treated with bis-(1,3-dibutylbarbituric acid) trimethine oxonol [DiBAC$_4$(3)] (Molecular Probes, Eugene, OR). Fluorescence intensity was measured using a FACSVerse flow cytometer (Becton Dickinson, USA) [8].

Propidium Iodide (PI) Uptake and Membrane Fluidity Measurement Using 1,6-Diphenyl-1,3,5-Hexatriene (DPH)

Cells were collected and suspended in PBS with periplanetasin-4 and its analogs at the minimum inhibitory concentration (MIC). After incubation for 2 h, the cells were harvested by centrifugation and resuspended in PBS. To assess membrane permeability, the cells were stained with 9 μM PI and incubated for 5 min at room temperature. Then, the cells were analyzed using a FACSVerse flow cytometer [15]. Fluorescence emitted from the plasma membrane of the bacterial cells labeled with DPH (Molecular Probes, USA) was used to monitor changes in membrane dynamics. Cells (2×10^7 cells/ml) incubated with peptides for 2 h at 37°C were fixed with 0.37% formaldehyde. After washing with cold PBS, the cells were freeze-thawed with liquid nitrogen and washed with warm PBS twice. The suspensions were incubated with 0.6 mM DPH for 45 min at 37°C and washed with PBS three times. The fluorescence intensity of DPH was measured using a spectrofluorophotometer at 350/425 nm (excitation/emission) [16].

Preparation of Large Unilamellar Vesicle Liposomes

Large unilamellar vesicles (LUVs) were prepared as described previously [7]. The desired phospholipid mixture (phosphatidylethanolamine:phosphatidylglycerol=3:1) [17] was dissolved in chloroform and dried in a round glass flask under argon gas. The encapsulated dye was comprised of calcein and fluorescein isothiocyanate-labeled dextrans (FDs) with average molecular weights of 4000 and 10000. All FDs were purchased from Sigma: calcein (molecular weight (mw), 623 Da; Stokes-Einstein radius = 0.74 nm), FD4 (mw, 3.9 kDa; Stokes-Einstein radius = 1.4 nm) and FD10 (mw, 9.9 kDa; Stokes-Einstein radius = 2.3 nm) [18].

The suspensions were subjected to 13 freeze-thaw cycle and extruded through two stacked polycarbonate filters (200-nm pores) with a LiposoFast extruder (Avestin Inc., Canada). Gel filtration chromatography on a Sephadex G-50 column was performed to separate LUVs from un-capsulated dye. Leakage of capsulated calcein or FDs from the liposome was monitored by measuring the intensity of fluorescence at an excitation wavelength of 494 nm and an emission wavelength of 520 nm using a spectrofluorophotometer (Shimadzu, RF-5301PC, Shimadzu, Japan). Triton X-100 (0.1%) was used to determine 100% dye release. The percentage of dye leakage (%) = $(F\text{-}F_0)/(F_t\text{-}F_0) \times 100$. *F* represents the fluorescence intensity achieved after addition of the peptides. F_0 and F_t represent the fluorescence intensities of untreated samples and those treated with Triton X-100, respectively.

Estimation of the Pore Size in Artificial Membrane

To evaluate the pore sizes of periplanetasin-4 and its analogs, the release of fluorescent dyes from the LUVs was monitored by measuring the fluorescence intensity at an excitation wavelength of 490 nm and an emission wavelength of 520 nm with a spectrofluorophotometer (Shimadzu, RF-5301PC, Shimadzu). Subsequently, soluble fluorescent molecules, including calcein, FD4 and FD10, were included to a final concentration of 2 μM, 0.1 mg/ml, and 0.1 mg/ml, respectively. All FDs were purchased from Sigma Chemical Co. (USA). The percentage of dye leakage caused by the peptides was calculated as follows: leakage (%) = $100 \times (F\text{-}F_0)/(F_t\text{-}F_0)$, where F represents the fluorescence intensity achieved after addition of the peptides and F_0 and F_t represent the fluorescence intensities without the compounds and with Triton X-100, respectively [8]. Triton X-100 was added to obtain 100% leakage.

Statistical Analysis

Values are reported as the mean ± standard deviation (SD) from three independent experiments. Statistical significance was determined using Student's *t*-test. Differences between the samples were considered to be significant at *p*-values < 0.05, < 0.01, and < 0.001.

Results and Discussion

Peptide Design and Synthesis of Periplanetasin-4 Analogs

An ideal AMP-based drug would have programmable specificity for selectively attacking the targeted cell or cell membrane [19]. Most AMPs have a high net positive charge resulting from lysine and arginine residue [9]. To improve the antimicrobial activity, the positive charge of the peptides was increased by replacing neutral and acidic amino acids with cationic amino acids such as arginine or lysine. By doing so, the electrostatic interaction between the cell membranes and AMPs could be reinforced [9]. In our previous study, periplanetasin-4 was identified from American cockroaches as an AMP. The peptide contains 13 amino acids, including one hydrophilic arginine residue and one cysteine residue [8]. Periplanetasin-4 shows not only cationic but also amphipathic properties. Various parameters, including net positive charge, overall hydrophobicity, and helicity have been shown to modulate the antimicrobial activity of the amphipathic AMPs [20, 21]. To estimate the structural/functional role of periplanetasin-4, we designed and synthesized three analogs on the basis of the boundary between the hydrophobic and charged regions of the peptide (Fig. 1). The cysteine, tyrosine, and histidine in perplanetasin-4 were replaced with the positively charged arginine [22]. Arginine is the most positively

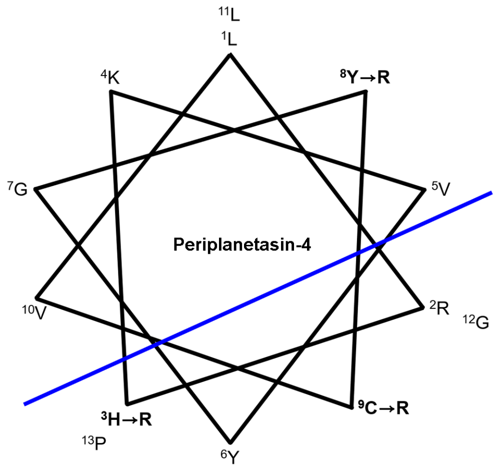

Fig. 1. Helical wheel diagram of the analogs of periplanetasin-4. The diagram is drawn at a rotation angle of 108 degrees per peptide bond [36]. The oblique line indicates the boundary between the hydrophobic residues and the charged/polar residues. The arrows indicate the amino acid residues substituted in their analog peptides.

charged amino acid and its pKa is 12.5. The number of arginine residues would modulate for efficient translocation [23]. In addition, arginine substitution can enhance net charge and reduce the hydrophobicity of peptides [22, 23]. Hydrogen-bond formation of the guanidino moiety in arginine with phosphates, carboxylates and sulfates in cellular components are proposed to be crucial for cell-permeation efficacy [23, 24]. Therefore, arginine substitution has become the design basis to create and enhance peptides with high cell-penetrating efficiency and activity [22]. The standard Fmoc solid-phase peptide was employed to synthesize wild-type periplanetasin-4 and its three analogs. The purity of the synthesized linear peptides was >90%, as measured by HPLC. The sequences of the synthetic peptides are shown in Table 1. We calculated hydrophobicity by using the Eisenberg–Weiss scale and determined that Anal-1, Anal-2, and Anal-3 had values of -2.67, -4.49, and -5.89, respectively, while periplanetasin-4 had a value of -0.83 (Table 1). Additionally, the relative hydrophobicity of the analogs could be determined by measuring their retention times on a reverse-phase HPLC C_{18} column. Peptide hydrophobicity can be computed as the sum of the hydrophobicity values (retention coefficients) of the constituent amino acids [25]. The decrease in the retention time of the analogs seems to be similar to the calculated hydrophobic property. The substitution from tyrosine, cysteine, and histidine leads to changes in the functional group consisting of a 3-carbon aliphatic straight chain ending in a guanidino group to tyrosyl, thiol, and imidazole, respectively [9, 11]. Arginine has the highest hydrophilicity, according to the Eisenberg and Weiss calculation. Despite possessing a polar sulfhydryl group, cysteine in folded protein structures behaves as a hydrophobic residue rather than a polar residue [26]. The presence of tyrosine imparts hydrophobic properties due to the tyrosyl group, albeit to a lower degree than cysteine [27]. A histidine imidazolyl ring results in a hydrophilic nature, but has stronger hydrophobicity than arginine [28]. These changes bring about a decrease in hydrophobicity [9, 11].

Antibacterial and Hemolytic Activities of Periplanetasin-4 and Its Analogs

To explore whether the substitution altered the antibacterial activity, a bacterial susceptibility test was performed. The antibacterial and hemolytic activities of periplanetasin-4 were evaluated against a representative set of pathogenic gram-positive and gram-negative bacteria. Generally, an increase in net charge affects the activity of AMPs due to interactions with negatively charged bacterial membranes [9, 22, 23]. As shown in Table 2, all bacterial strains were susceptible to periplanetasin-4 and its analogs. Although periplanetasin-4 showed the most potent activities, its analogs also showed antibacterial activity. As shown in Table 2, all bacterial strains were susceptible to periplanetasin-4 and its analogs. Although periplanetasin-4 showed the most potent activity, its analogs also showed notable antibacterial activities. Anal-1 had MIC values ranging

Table 1. Amino acid and physicochemical properties of periplanetasin-4 and analogs.

Peptides	Amino acid sequence	Substitution	Molecular mass (Da)	Net charge (physiological pH)	Retention time (min)	Hydrophobicity
Periplanetasin-4	1 … 10<br>LRHKVYGYCVLGP-**NH$_2$**	Native	1503.8	+3.0	19.975	-0.83
Anal-1	LRHKVYGY**R**VLGP-**NH$_2$**	$C^9 \rightarrow R^9$	1556.88	+4.1	14.419	-2.67
Anal-2	LRHKVYG**RR**VLGP-**NH$_2$**	$Y^8C^9 \rightarrow R^8R^9$	1549.89	+5.1	12.644	-4.49
Anal-3	LR**R**KVYG**RR**VLGP-**NH$_2$**	$H^3Y^8C^9 \rightarrow R^3R^8R^9$	1568.94	+6.1	12.646	-5.89

Table 2. The antimicrobial activity of periplanetasin-4 and its analogs.

Microbial strains	MIC (μM)			
	Periplanetasin-4	Anal-1	Anal-2	Anal-3
Gram-positive bacteria				
Enterococcus faecium ATCC 19434	2.5-5	5	5	10
Enterococcus faecalis ATCC 29212	2.5	5	10	10
Staphylococcus epidermidis KCTC 1917	2.5	2.5-5	5	5-10
Streptococcus mutans KCTC 3065	2.5	5	10	20
Gram-negative bacteria				
Escherichia coli BW25113	2.5-5	5	5	10-20
Pseudomonas aeruginosa ATCC 27853	5	10	10	20
Salmonella typhimurim KCTC 1926	2.5	5	5-10	20
Salmonella enteritidis ATCC 13076	2.5	10	10	10-20

from 2.5−10 μM. Anal-2 had antibacterial properties in the 5−10 μM range and Anal-3 showed activity from 10−20 μM. For the analogs, the change in hydrophobicity is considered to be more crucial than the increase of net positive charge. Furthermore, to evaluate the hemolytic effect of the periplanetasin-4 analogs on human erythrocytes, their hemolytic activities were evaluated by measuring the amount of hemoglobin released from treated erythrocytes. The hemolytic effect of periplanetasin-4 on human erythrocytes was lower according to previous study [8]. At a high concentration of peptides (80 μM), periplanetasin-4 and all of its analogs exhibited hemolytic activity (Table S1). Likewise, the analogs of periplanetasin-4 maintained the cell selectivity toward bacterial cells without hemolysis. Hemolytic activity was enhanced by increasing hydrophobicity and amphipathicity and balancing peptide hydrophobicity and charge distribution promotes efficient antimicrobial activity without hemolytic activity [9, 29, 30]. There was no specific discrepancy between periplanetasin-4 and its analogs. These results suggest that the decrease in hydrophobicity at positions 3, 8, and 9 plays a significant role in the antimicrobial activities of periplanetasin-4 and the substitutions did not result in hemolysis. Optimization of the antimicrobial effect via modification of only the peptide charge may be limited [9, 30]. These observations indicate that the attenuation of hydrophobic interactions rather than electrostatic interactions affect the antibacterial activity of periplanetasin-4, except for hemolysis.

Analogs of Periplanetasin-4 Induce a Decrease in Membrane Depolarization

Cationic properties are extremely important for binding to the negatively charged membranes of bacterial surfaces. The α-helical amphipathic propensity depends upon a proper balance between cationic, hydrophilic and hydrophobic residues and the propensity is a key factor for facilitating insertion into cell membranes and/or translocation across cell membranes, ultimately leading to cell death [9]. This mechanism depends on the stability of the bound lipid, favoring anionic headgroups such as that of phosphatidic acid, which thrive on the positive charge of the arginine [31]. On the other hand, modifying the peptide charge results in significant changes in one or more parameters, including hydrophobicity and amphipathicity, which are critical for the antimicrobial activity [9]. The amphipathic property can facilitate the effective interaction of the peptide with cell plasma membranes, which consist of phospholipids. Especially, periplanetasin-4 has been shown to exert its antifungal activity via interactions with the plasma membrane [8]. To examine whether the substitution of arginine affects the inherent bacterial membrane function, the changes in membrane potential were investigated. The action on the membranes can often lead to the collapse of the membrane electrochemical gradients [11]. The membrane depolarization was investigated using $DiSC_3(5)$ and $DiBAC_4(3)$. The addition of periplanetasin-4 resulted in an increase of $DiSC_3(5)$ fluorescence intensity, indicating membrane depolarization (Fig. 2). Meanwhile, the three analogs of periplanetasin-4 induced a moderate increase in fluorescence intensity compared to the increase of fluorescence intensity by periplanetasin-4. These patterns in the membrane depolarization mostly corresponded to the results of the antimicrobial susceptibility test, suggesting that it was difficult for the analogs to bind to the plasma membrane via electrostatic interactions.

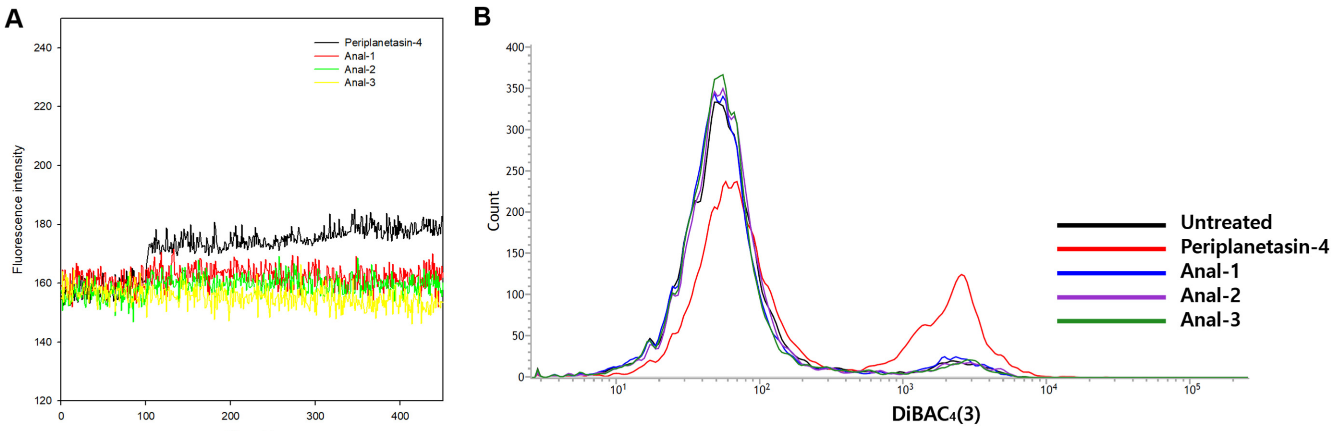

Fig. 2. Impact of periplanetasin-4 analogs on cell membrane function of *E. coli.*

(**A**) Depolarization of the membrane potential by DiSC$_3$(5) was detected. DiSC$_3$(5) was added at t = 30 s. After internalization of the probe, at t = 180 s, MIC of peptides was added to monitor changes in fluorescence (Ex. 622 nm and Em. 670 nm). (**B**) Flow cytometric analysis of DiBAC$_4$(3)-stained *E. coli* following incubation at the MIC of the peptides

Analogs of Periplanetasin-4 Diminish Membrane Disruptive Action

To further elucidate the dissimilarity of the mechanisms employed by periplanetasin-4 and its analogs, the membrane permeability and dynamics were investigated using PI and DPH. Periplanetasin-4-treated cells showed an accumulation of PI, indicating membrane permeabilization [16]. In contrast, cells treated with the analogs did not show PI uptake. Furthermore, the fluorescence intensity of DPH decreased in periplanetasin-4-treated cells compared to that in control untreated cells and the cell membrane became more unstable after peptide treatment (Fig. 2). This decrease in DPH intensity revealed the perturbation of the cell membrane following periplanetasin-4 treatment. The membrane alteration induced by the analogs was more attenuated than that induced by periplanetasin-4. The degree of membrane disruptive action gradually decreased in accordance with the arginine substitutions. On the basis of these results, arginine substitution reduced the ability of periplanetasin-4 to induce membrane permeabilization and decreased the membrane dynamics. The antimicrobial activities of cationic α-helical AMPs are governed by the positive charges and hydrophobicity of the residues [9]. The membrane disruptive action of the arginine-substituted analogs of periplanetasin-4 was reduced by the changes in their hydrophobicity and amphipathic propensity. Arginine provides a positive charge, facilitating binding to negatively charged bacterial components, such as lipopolysaccharide

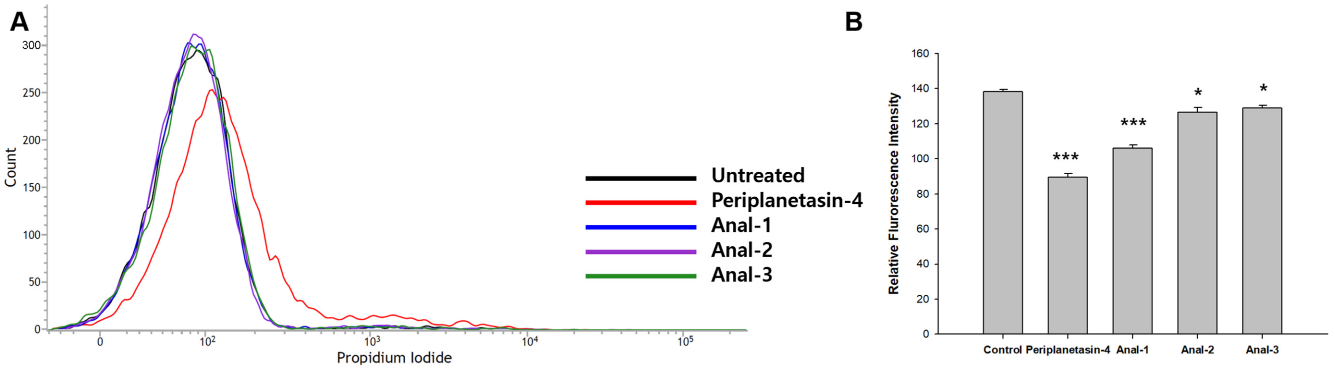

Fig. 3. Membrane permeabilization and dynamics of analogs of periplanetasin-4.

(**A**) Flow cytometric analysis of membrane permeabilization by PI staining. Cells were treated with periplanetasin-4 and its analogs for 2 h, then incubated with 9.0 μM PI. (**B**) DPH fluorescence intensity of cells treated with periplanetasin-4 and it analogs. Data are the means ± SD of three independent experiments. *$p < 0.05$; **$p < 0.01$; and ***$p < 0.001$ (vs. the control; Student's *t*-test).

and phosphatidylglycerol, which results in the increasing accumulation of AMPs on the membrane surface [32]. Additionally, arginine gives the cationic hydrophilic tail its charge and may be involved in peptide-lipid interactions. Nonetheless, a hydrophilic tail with cationic properties from arginine maintains the amphipathic peptide structure [33]. It seems that the decrease of hydrophobicity by substituting arginine dispersed the hydrophobic interactions between the hydrophobic residues of periplanetasin-4 and the hydrophobic tail region of the bacterial membranes before interactions between the positively charged amino acids and negatively charged components. This further suggests that the substitution to arginine could be attenuated by interactions between negatively charged phospholipids and peptides. It further suggests that the substitution to arginine could be attenuated by the interaction between the peptide and the negatively charged phospholipid. When considering the pattern of hydrophobic interactions, this observation suggested that hydrophobicity is one of the most crucial factors for the inhibition of cell survival.

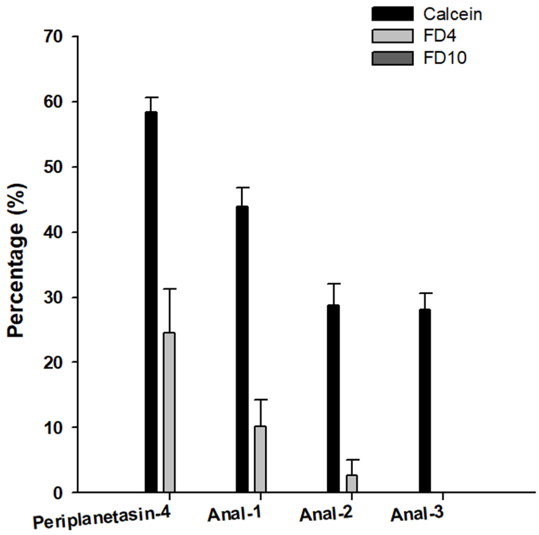

Fig. 4. Analysis of periplanetasin-4 interaction with liposomes. Percentage of calcein, FD4 and FD10 leaked from liposomes containing the lipid composition of *E. coli*, following incubation with periplanetasin-4 or its analogs. None of the peptides had any FD10 leakage.

Influence on Bacterial Artificial Membrane and Size of the Pores Formed by Periplanetasin-4 and Its Analogs

To further estimate the membrane damage caused by the analogs, an artificial membrane model was prepared and the release of several calcein and FD molecules from the liposome was measured. Encapsulated calcein molecules in phosphatidylethanolamine:phosphatidylglycerol (3:1, w/w) LUV were exhausted in all of the peptides. The hydrodynamic radius of calcein, FD4 and FD10 are known to be 0.74, 1.4, and 2.3 nm [34]. The sizes of the pores formed in the artificial plasma membrane treated with periplanetasin-4 were estimated to range from 1.4 to 2.3 nm [35]. Such damages, in addition to promoting the leakage of the cytoplasm components, lead to membrane depolarization, loss of membrane functions, homeostasis impairment, and ultimately the loss of cell viability and an imbalance in the intracellular ionic gradient [35]. However, the analogs did not interrupt the liposome and all of the calcein-release values appeared to be similar. These results indicated that the damage caused by the analogs did not destabilize the artificial membranes. The damage from each analog was small, ranging up to 0.74 nm (Fig. 4) [34]. Membrane disruption was not observed in the cells or the artificial membranes. In this case, the substituted arginine did not lead to an increase in the antibacterial activity although the positive net charge was increased. Consequentially, this result indicated that the substitution with three arginines decreased the hydrophobicity of the peptide, attenuating the interaction with the peptide and the membrane components. This indicated that the high affinity for hydrophobic interactions relates to the induction of membrane-targeted interactions. Hydrophobic interaction is one of the factors that contribute to membrane disruption by periplanetasin-4 rather than direct electrostatic interactions between the arginine residue and biomembranes. Although membrane disruptive action was drastically attenuated by substituting arginine, the analogs involved enough antibacterial activity to induce cell death. Combined with the results of previous studies, we speculated that periplanetasin-4 could target intracellular components [8] and hydrophobicity could be induced on the membrane. These observations will guide future design efforts to produce more active antimicrobial peptides.

Acknowledgement

This work was supported by a grant from the Next-Generation BioGreen 21 Program (PJ01325603), Rural Development Administration, Republic of Korea.

Conflict of Interest

The authors have no financial conflicts of interest to declare.

References

1. Chowanski S, Adamski Z, Lubawy J, Marciniak P, Pacholska-Bogalska J, Slocinska M, *et al.* 2017. Insect peptides - perspectives in human diseases treatment. *Curr. Med. Chem.* **24:** 3116-3152.
2. Smetana S, Palanisamy M, Mathys A, Heinz V. 2016. Sustainability of insect use for feed and food: life cycle assessment perspective. *J. Clean. Prod.* **137:** 741-751.
3. Yi HY, Chowdhury M, Huang YD, Yu XQ. 2014. Insect antimicrobial peptides and their applications. *Appl. Microbiol. Biotechnol.* **98:** 5807-5822.
4. La Barbera F, Verneau F, Amato M, Grunert K. 2018. Understanding Westerners' disgust for the eating of insects: the role of food neophobia and implicit associations. *Food Qual. Prefer.* **64:** 120-125.
5. Lee H, Hwang JS, Lee DG. 2016. Scolopendin 2 leads to cellular stress response in *Candida albicans*. *Apoptosis* **21:** 856-865.
6. Kim I-W, Lee JH, Subramaniyam S, Yun E-Y, Kim I, Park J, *et al.* 2016. De novo transcriptome analysis and detection of antimicrobial peptides of the American cockroach *Periplaneta americana* (Linnaeus). *PLoS One* **11:** e0155304.
7. Lee H, Hwang JS, Lee J, Kim JI, Lee DG. 2015. Scolopendin 2, a cationic antimicrobial peptide from centipede, and its membrane-active mechanism. *Biochim. Biophys. Acta* **1848:** 634-642.
8. Lee H, Hwang JS, Lee DG. 2019. Periplanetasin-4, a novel antimicrobial peptide from the cockroach, inhibits communications between mitochondria and vacuoles. *Biochem. J.* **476:** 1267-1284.
9. Taniguchi M, Ochiai A, Takahashi K, Nakamichi SI, Nomoto T, Saitoh E, *et al.* 2016. Effect of alanine, leucine, and arginine substitution on antimicrobial activity against candida albicans and action mechanism of a cationic octadecapeptide derived from alpha-amylase of rice. *Biopolymers* **106:** 219-229.
10. Roncevic T, Vukicevic D, Krce L, Benincasa M, Aviani I, Maravic A, *et al.* 2019. Selection and redesign for high selectivity of membrane-active antimicrobial peptides from a dedicated sequence/function database. *Biochim. Biophys. Acta Biomembr.* **1861:** 827-834.
11. Lee J, Choi H, Cho J, Lee DG. 2011. Effects of positively charged arginine residues on membrane pore forming activity of Rev-NIS peptide in bacterial cells. *Biochim. Biophys. Acta* **1808:** 2421-2427.
12. Uppu DS, Samaddar S, Ghosh C, Paramanandham K, Shome BR, Haldar J. 2016. Amide side chain amphiphilic polymers disrupt surface established bacterial bio-films and protect mice from chronic *Acinetobacter baumannii* infection. *Biomaterials* **74:** 131-143.
13. Sun C, Li Y, Cao S, Wang H, Jiang C, Pang S, *et al.* 2018. Antibacterial activity and mechanism of action of bovine lactoferricin derivatives with symmetrical amino acid sequences. *Int. J. Mol. Sci.* **19(10).** pii: E2951
14. Rajasekaran G, Kim EY, Shin SY. 2017. LL-37-derived membrane-active FK-13 analogs possessing cell selectivity, anti-biofilm activity and synergy with chloramphenicol and anti-inflammatory activity. *Biochim. Biophys. Acta Biomembr.* **1859:** 722-733.
15. Cherrat L, Dumas E, Bakkali M, Degraeve P, Laglaoui A, Oulahal N. 2016. Effect of essential oils on cell viability, membrane integrity and membrane fluidity of *Listeria innocua* and *Escherichia coli*. *J. Essent. Oil-Bear. Plants* **19:** 155-166.
16. Lee H, Woo ER, Lee DG. 2016. (-)-Nortrachelogenin from *Partrinia scabiosaefolia* elicits an apoptotic response in *Candida albicans*. *FEMS Yeast Res.* **16(3).** pii: fow013
17. Malanovic N, Lohner K. 2016. Gram-positive bacterial cell envelopes: the impact on the activity of antimicrobial peptides. *Biochim. Biophys. Acta (BBA)-Biomembr.* **1858:** 936-946.
18. Leclercq SY, Sullivan MJ, Ipe DS, Smith JP, Cripps AW, Ulett GC. 2016. Pathogenesis of Streptococcus urinary tract infection depends on bacterial strain and beta-hemolysin/cytolysin that mediates cytotoxicity, cytokine synthesis, inflammation and virulence. *Sci. Rep.* **6:** 29000.
19. Mayer SF, Ducrey J, Dupasquier J, Haeni L, Rothen-Rutishauser B, Yang J, *et al.* 2019. Targeting specific membranes with an azide derivative of the pore-forming peptide ceratotoxin A. *Biochim. Biophys. Acta Biomembr.* **1861:** 183023.
20. Lee DG, Kim HN, Park Y, Kim HK, Choi BH, Choi C-H, *et al.* 2002. Design of novel analogue peptides with potent antibiotic activity based on the antimicrobial peptide, HP (2–20), derived from N-terminus of Helicobacter pylori ribosomal protein L1. *Biochim. Biophys. Acta* **1598:** 185-194.
21. Huang Y, Huang J, Chen Y. 2010. Alpha-helical cationic antimicrobial peptides: relationships of structure and function. *Protein Cell* **1:** 143-152.
22. Zhang P, Ma J, Yan Y, Chen B, Liu B, Jian C, *et al.* 2017. Arginine modification of lycosin-I to improve inhibitory activity against cancer cells. *Org. Biomol. Chem.* **15:** 9379-9388.
23. Tada N, Horibe T, Haramoto M, Ohara K, Kohno M, Kawakami K. 2011. A single replacement of histidine to arginine in EGFR-lytic hybrid peptide demonstrates the improved anticancer activity. *Biochem. Biophys. Res. Commun.* **407:** 383-388.
24. Nakase I, Takeuchi T, Tanaka G, Futaki S. 2008. Methodological and cellular aspects that govern the internalization mechanisms of arginine-rich cell-penetrating peptides. *Adv. Drug Deliv. Rev.* **60:** 598-607.
25. Krokhin O. 2012. Peptide retention prediction in reversed-phase chromatography: proteomic applications. *Expert Rev. Proteomics* **9:** 1-4.

26. Iyer BR, Mahalakshmi R. 2019. Hydrophobic characteristic is energetically preferred for cysteine in a model membrane protein. *Biophys. J.* **117:** 25-35.
27. Bartesaghi S, Herrera D, Martinez DM, Petruk A, Demicheli V, Trujillo M, *et al.* 2017. Tyrosine oxidation and nitration in transmembrane peptides is connected to lipid peroxidation. *Arch. Biochem. Biophys.* **622:** 9-25.
28. El-Sayed N, Miyake T, Shirazi A, Park S, Clark J, Buchholz S, *et al.* 2018. Design, synthesis, and evaluation of homochiral peptides containing arginine and histidine as molecular transporters. *Molecules* **23:** 1590.
29. Lee TH, Hall KN, Aguilar MI. 2016. Antimicrobial peptide atructure and mechanism of ction: a focus on the role of membrane structure. *Curr. Top Med. Chem.* **16:** 25-39.
30. Zhang S-K, Song J-w, Gong F, Li S-B, Chang H-Y, Xie H-M, *et al.* 2016. Design of an α-helical antimicrobial peptide with improved cell-selective and potent anti-biofilm activity. *Sci. Rep.* **6:** 27394.
31. Poveda JA, Giudici AM, Renart ML, Millet O, Morales A, Gonzalez-Ros JM, *et al.* 2019. Modulation of the potassium channel KcsA by anionic phospholipids: Role of arginines at the non-annular lipid binding sites. *Biochim. Biophys. Acta Biomembr.* **1861:** 183029.
32. Yu L, Fan Q, Yue X, Mao Y, Qu L. 2015. Activity of a novel-designed antimicrobial peptide and its interaction with lipids. *J. Pept. Sci.* **21:** 274-282.
33. Park C, Cho J, Lee J, Lee DG. 2011. Membranolytic antifungal activity of arenicin-1 requires the N-terminal tryptophan and the beta-turn arginine. *Biotechnol. Lett.* **33:** 185-189.
34. Sandoval CM, Salzameda B, Reyes K, Williams T, Hohman VS, Plesniak LA. 2007. Anti-obesity and anti-tumor pro-apoptotic peptides are sufficient to cause release of cytochrome c from vesicles. *FEBS Lett.* **581:** 5464-5468.
35. Araújo NM, Dias LP, Costa HP, Sousa DO, Vasconcelos IM, de Morais GA, *et al.* 2019. ClTI, a Kunitz trypsin inhibitor purified from Cassia leiandra Benth. seeds, exerts a candidicidal effect on *Candida albicans* by inducing oxidative stress and necrosis. *Biochim. Biophys. Acta Biomembr.* **1861(11):** 183032.
36. Kubota S, Pomerantz RJ. 1998. A cis-acting peptide signal in human immunodeficiency virus type I Rev which inhibits nuclear entry of small proteins. *Oncogene* **16:** 1851-1861.
